# Right-side fixation of the sigmoid colon causing internal herniation with closed-loop obstruction of both small and large bowel: a case report and review of the literature

**DOI:** 10.1186/s13256-022-03529-x

**Published:** 2022-08-30

**Authors:** G. Bertelli, S. Patauner, T. Gorgatti, A. Frena

**Affiliations:** 1 Department of General Surgery, Bolzano Central Hospital, South Tyrol, Italy; 2 Department of Neuroradiology, Bolzano Central Hospital, South Tyrol, Italy

**Keywords:** Right-side fixation of the sigmoid colon, Internal hernia, Intestinal malrotation, Closed-loop obstruction, Renal ectopia, Case report

## Abstract

**Background:**

Right-side fixation of the sigmoid colon is a rare anatomical variant associated with intestinal malrotation (Choi *et al*. in J Korean Surg Soc. 84(4):256–60, 2013). Differently from other forms of malrotation, this variant has not been associated thus far with acute surgical conditions.

**Case presentation:**

In this report, we present a 65-year-old Caucasian patient admitted for bowel obstruction symptoms. Computed tomography scan revealed right-side fixation of the sigmoid colon extended to the subhepatic recess complicated by obstructed internal herniation of the ileum. In this patient, the sigmoid colon occupied a recess posterior to the ascending colon and right Toldt’s fascia. Within this narrow anatomical space, an ileal loop was trapped causing internal herniation with resultant close-bowel obstruction of both ileum and sigmoid colon. The ileal loop was released surgically and the anatomical abnormality corrected.

**Conclusions:**

To our knowledge, this is the first case of right-side fixation of the sigmoid colon causing acute obstruction secondary to internal herniation of the small intestine. Early recognition and precise anatomical definition of such anatomical variants are essential to optimize their surgical approach.

## Introduction

Right-side fixation of sigmoid colon is a rare anatomical condition where the sigmoid colon is fixed on the right posterior abdominal wall. It is often associated with dolichosigma and with various forms of intestinal malrotation [1]. Only 12 such cases have been reported thus far [1, 3–9]. In the majority of these, the sigmoid colon was fixed in the right iliac fossa with only few cases describing fixation up to the subhepatic recess. This anatomical variant is most likely benign given that in all reports it represented an accidental finding during autopsies or during surgeries performed for other conditions. Nonetheless, in our case, right-side fixation was associated with obstructed internal herniation of the ileum likely due to the extension of fixation to the subhepatic recess with the creation of a narrow anatomical space.

## Case presentation

### Clinical history and physical examination

A 65-year-old Caucasian man was admitted to our hospital with a 12-hour history of diffuse colicky abdominal pain, nausea, and vomiting. The pain had arisen suddenly and presented a gross localization to the upper quadrants. He had failed to pass feces and gas for the last 2 days and reported no significant abdominal symptoms before the event except for mild stypsis. His past medical history included a previous thoracoscopic mitral valve repair and mild renal insufficiency secondary to ectopic hypoplastic left kidney located in the pelvis. He had no history of previous abdominal surgery. Upon physical examination, we found significant abdominal distension, severe spontaneous pain in the upper abdomen without guarding or positive Blumberg sign, and tympany on percussion. The body temperature was 37 °C, and all vital parameters were normal. Digital rectal examination revealed empty ampulla with normal fecal residues. A nasogastric tube was positioned, and about 400 ml of biliary effluent was drained.

### Diagnostic assessment

**Fig. 1:**
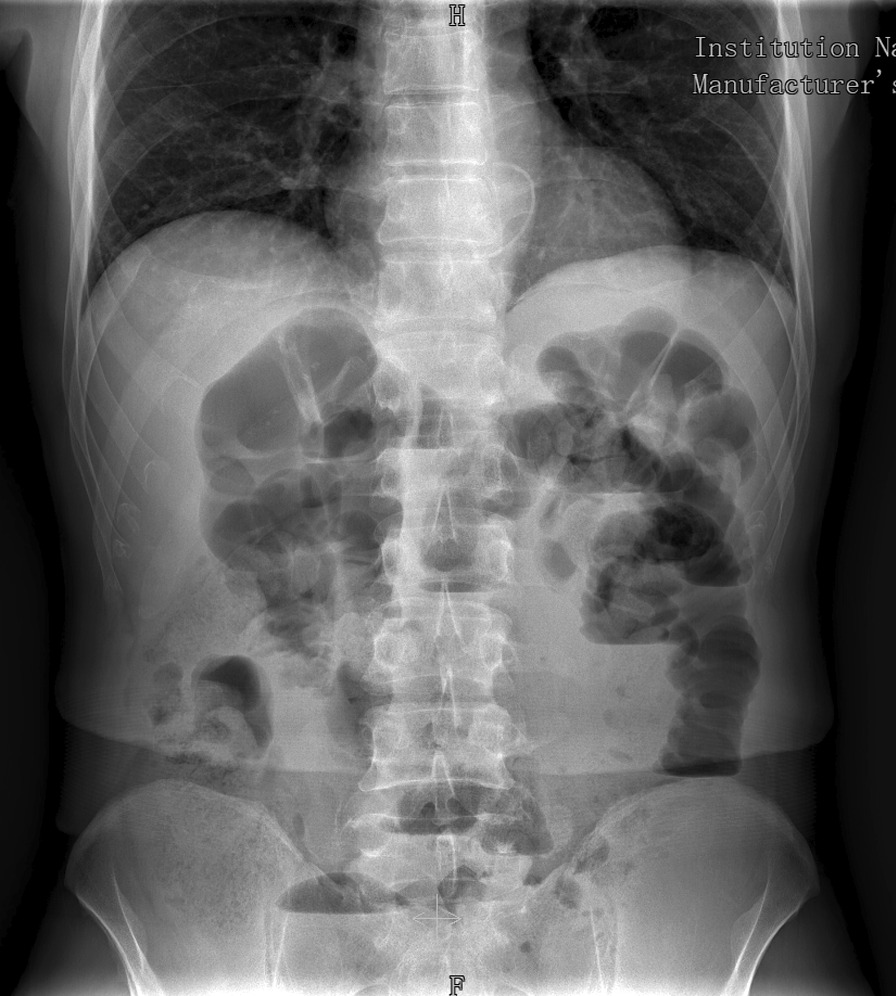
Abdominal X-ray

Blood tests revealed neutrophilia (leukocytes 9.98 × 103/μl; neutrophils 9.08 × 103/μl) and elevated C-reactive protein (CRP; 65 mg/L). Abdominal X-ray, in turn, showed diffuse abdominal distension of both small and large bowel with multiple air–fluid levels. The small bowel presented valvulae conniventes without mucosal thickening, while the large bowel showed coprostasis and regular haustration. Cecum diameter measured about 6 cm (Fig. 1).

**Fig. 2:**
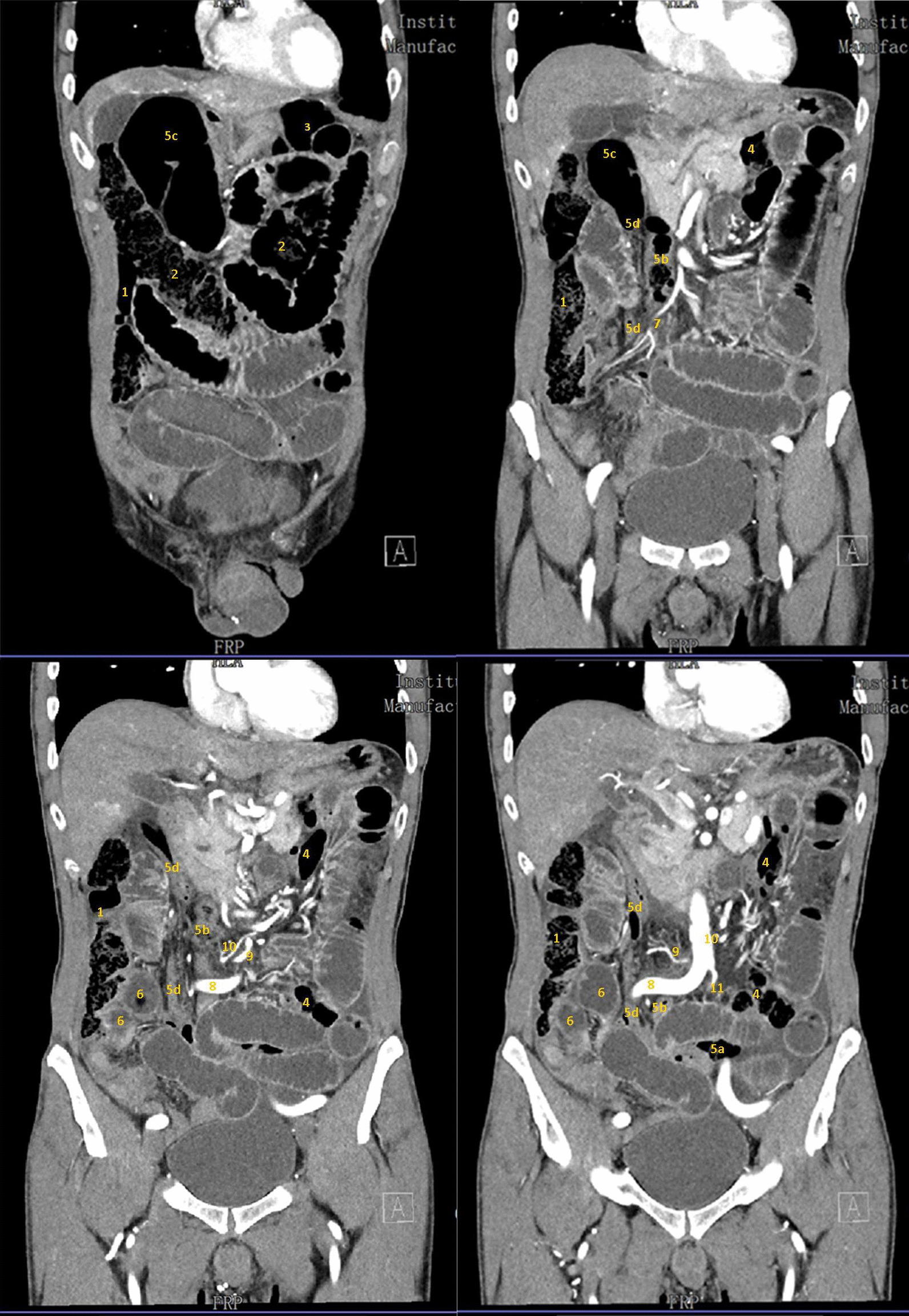
Coronal view of abdominal Computed tomography scan (from anterior to posterior): (1) ascending colon; (2) transverse colon; (3) splenic flexure; (4) descending colon; (5) sigmoid colon: a first, b second, c third, d fourth part; (6) incarcerated ileal loop; (7) ileocolic artery; (8) right common iliac artery; (9) inferior mesenteric artery; (10) left colic artery; (11) left renal artery

Abdominal contrast-enhanced computed tomography (CT) scan was thus performed showing an anatomical abnormality of the colon and diffuse dilation of both the small bowel and colon. The sigmoid colon was elongated and formed a distended loop in the subhepatic recess above the right flexure. Cecum and ascending colon were in their normal position, while the left ectopic kidney was found on the left side of the pelvis. Pancreas, duodenum, right kidney, celiac trunk, and superior mesenteric artery had no significant abnormalities (Fig. 2).

Importantly, the lack of left kidney bulk in the left flank altered the course of the colonic portions corresponding to the splenic flexure and descending segment. The splenic flexure was twisted counterclockwise on the frontal plane, causing the descending colon to be more medial and posterior. The latter therefore crossed posteriorly to the body of pancreas near the L2 vertebral body and descended anteriorly to the psoas muscle. From there onward, the course of the sigmoid colon could be divided into four segments. The first segment began in the left iliac fossa, turning right and crossing the midline at the level of the L5–S1 intervertebral disc near the upper pole of the left ectopic kidney. The second segment turned sharply upward directed to the subhepatic recess running anterior to the inferior vena cava and crossing the right common iliac artery anteriorly and the ileocolic vessels and proximal transverse colon posteriorly. The third segment formed a dilated loop in contact with the gallbladder and the lower aspect of the right hepatic lobe. The fourth segment continued in an inferior direction running along the right side of the inferior vena cava, crossing the right side of the promontory, and terminating in the rectum. Since the third part was dilated to 5 cm in diameter and the second and fourth parts were compressed, the sigmoid colon appeared to be involved in a closed-loop occlusion. A C-shaped fluid-filled loop of ileum with beak-like proximal and distal narrowing was found near the fourth part of sigmoid colon, resembling a closed-loop small bowel obstruction. Both the ileal loop and the sigmoid colon appeared to be incarcerated in a narrow space behind the ileocolic vessels.

**Fig. 3:**
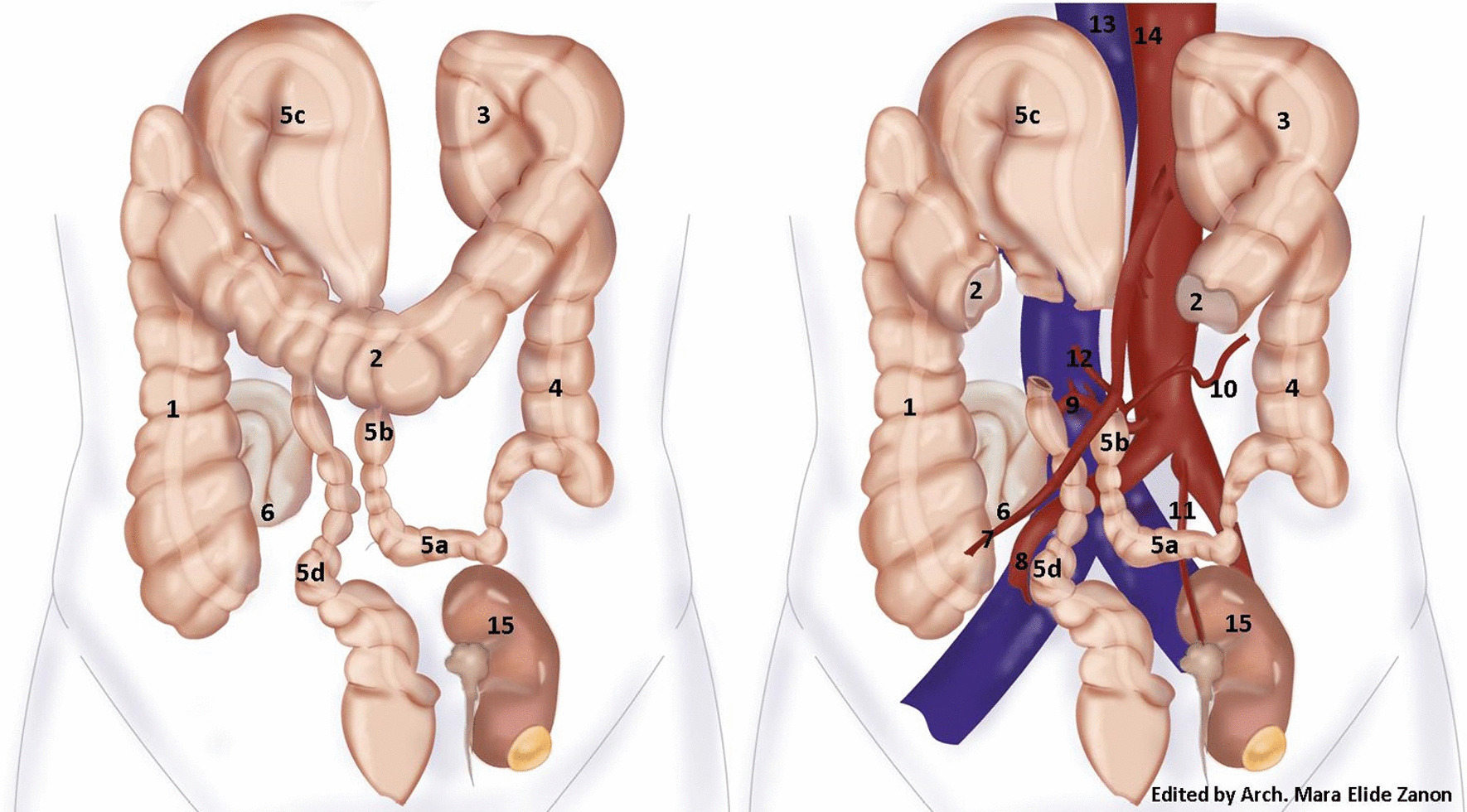
Schematic drawing of the anatomical abnormalities. (1) ascending colon; (2) transverse colon; (3) splenic flexure; (4) descending colon; (5) sigmoid colon: a first, b second, c third, d fourth part; (6) incarcerated ileal loop; (7) ileocolic artery; (8) right common iliac artery; (9) inferior mesenteric artery; (10) left colic artery; (11) left renal artery; (12) first sigmoid artery; (13) inferior vena cava; (14) aorta; (15) ectopic left kidney

The inferior mesenteric artery (IMA) was also characterized by an abnormal course. This originated from the aorta 2.5 cm above the aortic bifurcation and ran rightward, giving rise to a long recurrent left colic artery and some directed upward sigmoid branches. It then turned downward into the pelvis as the superior hemorrhoidal artery. The left colic artery had an extremely long course: it originated from the IMA via a common trunk with the first sigmoid artery to the right side of the aorta and crossed it running left and upward. The left renal artery originated on the anterior face of the aortic bifurcation and flowed downward to the ectopic kidney located on the left in the small pelvis (Fig. 3).

### Surgical intervention

**Fig. 4:**
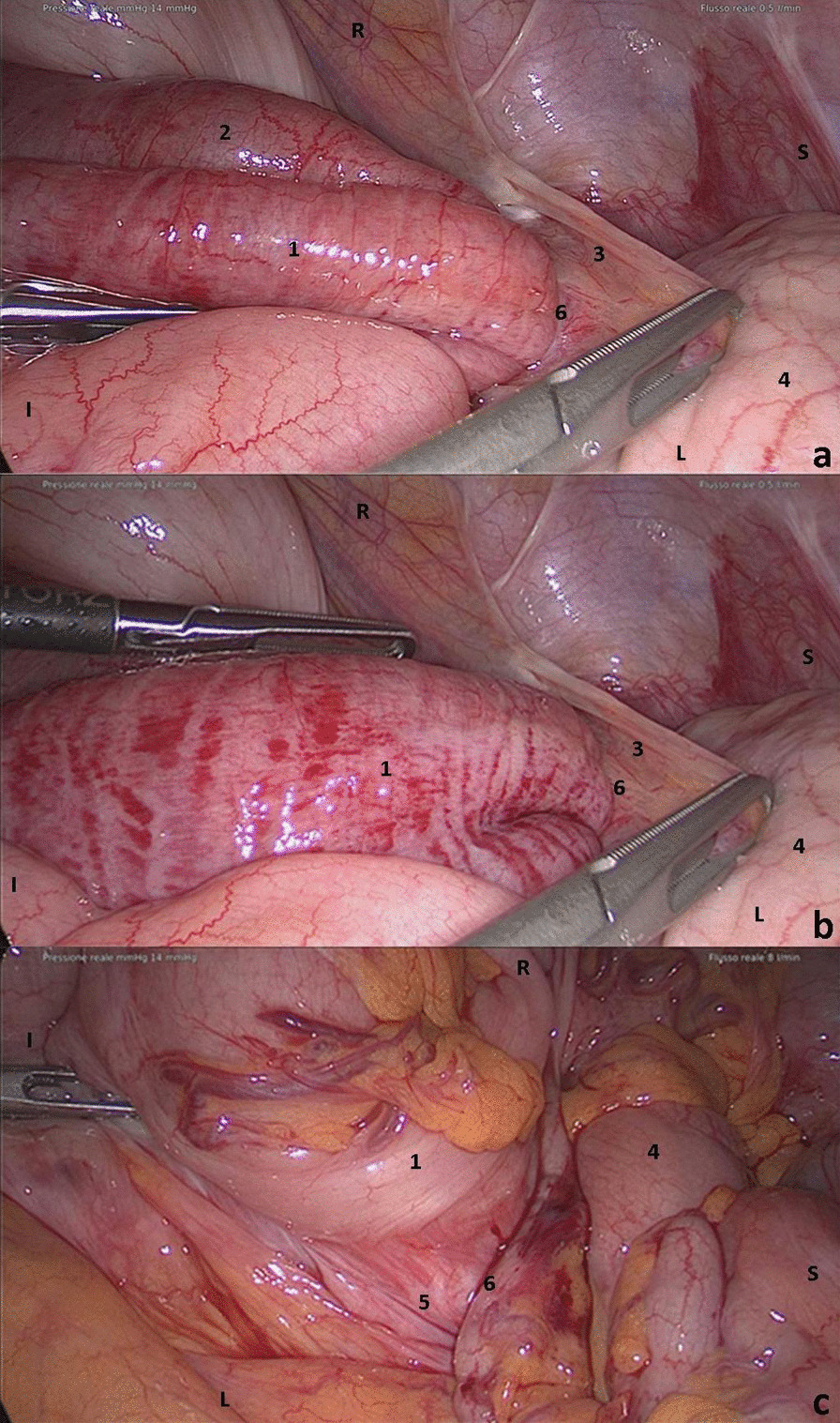
Intraoperative images: **a** ileal loop and sigmoid colon in the hernia breach. **b** Ileal loop was extracted from the hernia breach, and the sigmoid colon was carefully extracted. **c** Complete extraction of sigmoid colon shows fixation with the retroperitoneum (1) sigmoid colon; (2) incarcerated ileal loop; (3) adhesions between ascending colon and abdominal wall; (4) ascending colon; (5) adhesions between sigmoid colon and right peritoneum; (6) hernial breach. *S* cranial, *I* caudal, *L* left, *R* right

Given the complex anatomy, we opted for exploratory laparoscopy, which revealed a recess on the right posterior peritoneum traversed by the sigmoid colon. An ileal loop was trapped within this space, resulting in an internal hernia with upstream bowel dilation. This recess was limited anteriorly by Toldt’s fascia, the ascending colon and adhesions spanning between the ascending colon and the abdominal wall. These adhesions were similar to the Ladd’s bands described in malrotations. The ileal loop was extracted from the recess and did not appear ischemic, with deflation ensuing shortly after this procedure. The sigmoid colon was then extracted, revealing thick adhesions between the sigmoid colon and the posterior abdominal wall forcing the sigmoid colon to recoil inside the recess once traction from laparoscopic clamp was eased. Given the inability to safely lyse these adhesions, we decided to convert to open surgery. The adhesions of the sigmoid colon and its mesosigmoid with the retroperitoneum were partially lysed, allowing the sigmoid colon to remain in natural position without tension. Thereafter, a suture of the hernial breach was performed between the lower edge of Toldt’s fascia and the retroperitoneum. We did not perform complete lysis of sigmoid colon adhesions as this would have led to the complete mobilization of a dolichosigma with significant risk of volvulus. The procedure was completed with appendicectomy and positioning of abdominal drainage in the pelvis (Fig. 4).

### Postoperative course

The postoperative course was free of complications. Resumption of peristalsis was on the fifth postoperative day with re-alimentation beginning on the third postoperative day. The drainage was removed on the fifth postoperative day, and the entire hospital stay lasted 7 days. The patient did not report further symptoms except for the persistence of mild stypsis neither at postoperative day 12 nor at 6-month follow-up.

## Discussion

As highlighted in the anatomical description above, the patient presented with a right-side fixation of a prominent dolichosigma reaching the subhepatic recess and associated with some elements of malrotations, such as Ladd’s bands and lack of adhesion of the right colon to the retroperitoneum [2]. Despite the presence of these malrotation elements, the ascending colon was rotated normally given that the cecum was found in the right iliac fossa. The lack of adhesions between the sigmoid colon and the ascending colon resulted in a recess with a narrow neck predisposing to internal hernia. Therefore, the sigmoid colon and ileal loop trapped within this recess both developed closed-loop occlusion. Despite this peculiar anatomic condition, the patient remained asymptomatic for most of his life before the onset of the aforementioned complication.

To our knowledge, right-side fixation of sigmoid colon has been described in eight reports of about 12 individuals (either living or on autopsy) [1, 3–9]. In none of these cases was this condition associated with acute surgical conditions secondary to the anatomic abnormality. The fixed sigmoid colon was described mostly in the right iliac fossa [3, 4] or medially [5] to the right colon; however three cases of sigmoid colon posterior to the right colon have also been described [6, 7]. A right-side-fixed dolichosigma reaching the subhepatic recess was described in only two cases by Choi [1] and Komiyama [6]. However, only the case described by Komiyama appears to have anatomic features analogous to the present case. A course of the sigmoid colon below the mesenteric plane has also been described [10]; in this case, the sigmoid loop ran posterior to the root of the mesentery. Interestingly, while no specific link has been described with right-side fixation of the sigmoid colon, renal ectopia has been associated with intestinal malrotations [11]. Further in keeping with a potential link between these two conditions is the finding that left renal agenesis and ectopia are often associated with peculiar courses of the splenic flexure and descending colon [12].

Given that the present case represents an exceedingly rare finding with a very limited evidence base, surgical efforts were aimed at the resolution of the acute issue and the prevention of recurrence with minimal means. Repositioning of the sigmoid colon in its normal location was carried out mainly owing to the need for hernial breach closure between two firm structures such as Toldt’s fascia and retroperitoneum. On the other hand, we did not perform Ladd’s procedure (lysis of Ladd’s bands) as contact and duodenal stenosis were not found. Furthermore, while a complete lysis of Ladd’s bands may have constituted another valid approach allowing complete opening of the hernial recess, this would not have prevented the sigmoid colon to return to its previous position.

## Conclusion

This is the first reported case of internal obstructed hernia caused by a constellation of anatomic abnormalities associated with right-side fixation of the sigmoid colon. While these congenital anomalies of the colon are often asymptomatic and benign, they may predispose to acute complications such as obstructed internal hernias. In these instances, early recognition and precise anatomical definition are essential to optimize their surgical approach both to treat the acute complication and to prevent recurrence.

## Data Availability

Raw data were generated at Bolzano Central Hospital. Derived data supporting the findings of this study are available from the corresponding author BG on request.
